# A protease activated receptor-2 (PAR-2) activating peptide, tc-LIGRLO-NH_2_, induces protease release from mast cells: role in TNF degradation

**DOI:** 10.1186/1471-2210-4-12

**Published:** 2004-07-20

**Authors:** Hashem N Alshurafa, Grant R Stenton, John L Wallace, Morley D Hollenberg, A Dean Befus, Harissios Vliagoftis

**Affiliations:** 1 Glaxo-Heritage Asthma Research Laboratory, Pulmonary Research Group, Department of Medicine, Room 550A HMRC, University of Alberta, Edmonton, AB, Canada, T6G 2S2; 2 Department of Pharmacology & Therapeutics University of Calgary 3330 Hospital Drive NW Calgary AB, Canada T2N 4N1

## Abstract

**Background:**

Mast cell (MC)-derived serine proteases have been implicated in a variety of inflammatory processes. We have previously shown that rat peritoneal MC (PMC) express mRNA for protease activated receptor 2 (PAR-2), a G-coupled receptor activated by trypsin-like proteases. Recent evidence also suggests that MC-induced inflammation can be mediated through PAR. Therefore, we hypothesized that specific PAR-2 agonist peptides (PAR-2ap) induce protease release from PMC.

**Results:**

Western blot analysis of PMC supernatants revealed that a PAR-2ap, tc-LIGRLO (10 μM), stimulated the release of rat MC protease (RMCP)-1, RMCP-5 and carboxypeptidase-A. The release was evident by 20 min but further increased up to 8 h. To study the biological effects of protease release we tested supernatants from tc-LIGRLO, tc-OLRGIL (inactive control peptide) and antigen-activated PMC for proteolytic activity by seeding with TNF (150 pg/ml), incubating for 8 h at 37°C, and measuring TNF remaining in the supernatants. Supernatants from tc-LIGRLO-stimulated PMC degraded 44 % of seeded TNF (n = 5). Moreover, this TNF proteolysis was dependent on the concentration of tc-LIGRLO used to stimulate PMC, and was significantly inhibited (94 %) by soybean trypsin inhibitor. Antigen and tc-OLRGIL induced no significant release of such proteolytic activity.

**Conclusions:**

These data indicate that a PAR-2ap induces the release of proteases from mast cells, which may degrade extracellular cytokines and other substrates thus modulating the inflammatory response.

## Background

Protease activated receptor-2 (PAR-2) has been identified on a variety of cell types including eosinophils [1], neutrophils [2], neurons and smooth muscle cells [3]. It can be activated by a variety of serine proteases including MC tryptase [4], pancreatic trypsin [5], and coagulation factors [6] to induce inflammatory, mitogenic and chemotactic functions. Serine proteases cleave PAR-2 at a specific site in the extracellular NH_2_-terminus unmasking a new NH_2_-terminus (tethered ligand) and changing the conformation of the receptor to allow the tethered ligand to interact with the activation site on the 2^nd ^extracellular loop of the receptor. Peptides that are similar in sequence to the tethered ligand domains of PAR-2, such as SLIGRL-NH_2 _(SLI) or tc-LIGRLO-NH_2 _(tc-LIG) are able to interact directly with the activation site and act as potent agonists [7].

A growing number of studies have identified a role for PAR-2 in inflammation. There is a delayed onset of inflammation in PAR-2 knock out mice [8], and PAR-2-activating peptides (PAR-2ap) stimulate leukocyte rolling, adherence, and recruitment in rat mesenteric postcapillary venules [9]. Furthermore, PAR-2 activation of human airway epithelial cells mediates the release of the eosinophil survival-promoting factor GM-CSF and matrix metalloproteases [10,11].

The ability of serine proteases to activate MC and the observation that MC express PAR-2 [12,13], suggest that PAR-2-induced proinflammatory functions *in vivo *could be MC-mediated. The administration of PAR-2ap or trypsin into the rat hind paw enhanced vascular permeability and caused edema formation, which can be abolished by repeated pre-treatment with compound 48/80, known to deplete the MC of its granular content [14]. By contrast, Vergnolle *et al*., 1999 [15] showed that edema induced by injection of PAR-2ap was only slightly reduced in rats pre-treated with compound 48/80, and the pre-treatment of rats with cromolyn, a MC stabilizer, had no effects on PAR-2ap induced inflammation of the paw. These studies showed that the administration of PAR-2ap induces an acute inflammatory response characterized by persistent edema and granulocyte infiltration, but the involvement of MC in these responses requires further investigation.

Activation of PAR-2 on the surface of mast cells could act as part of an autocrine and paracrine positive feedback loop through the release of serine proteases that could activate further PAR-2 on mast cells or other neighboring cells. Therefore, we investigated the direct effects of PAR-2ap on the release of serine proteases from purified PMC and the effects of these released proteases on extracellular protein degradation. In particular we studied the release of rat mast cell protease-1 (RMCP-1), RMCP-5 and carboxypeptidase A (CPA).

## Results

### The PAR-2ap, tc-LIG, induces release of RMCP-1, RMCP-5 and CPA from PMC

**Figure 1:**
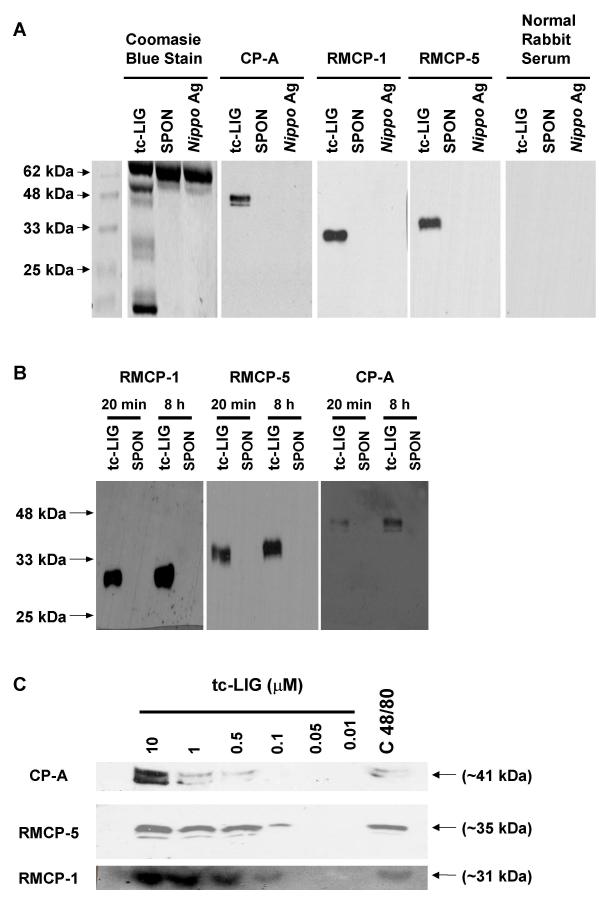
Release of RMCP-1, -5 and CPA from PMC following activation with tc-LIG (PAR-2ap), compound 48/80 and Ag. (A) Supernatants from tc-LIG (10 μM), Ag (10 We/mL) and sham-treated (spon) mast cells were concentrated (10 ×) and Western blot analysis preformed for CPA, RMCP-1 and RMCP-5. Left panel shows Coomassie blue staining of the same gel and right panel Western blot with normal rabbit serum as a negative control. (B) Release of RMCP-1, RMCP-5 and CPA following 20 min and 8 h activation of PMC with tc-LIG (10 μM). (C) Dose response for the release of RMCP-1, -5 and CPA by tc-LIG-stimulated or compound 48/80-stimulated PMC. In all cases representative blots from three experiments with similar results are shown.

To identify proteases released by mast cells following PAR-2ap stimulation we activated PMC with tc-LIG (10 μM), and analyzed the supernatants for various mast cell proteases by western blotting, using antisera against the amino-terminal sequences of RMCP-5 and MC-CPA and an antiserum against RMCP-1 protein. In supernatants from tc-LIG-treated PMC one band for RMCP-1 (30 kDa), two bands for RMCP-5 (34 and 35 kDa), and three bands for CPA (40, 41 and 42 kDa) were detected (Fig. 1A). The PAR-2ap tc-LIG induced most of the protease release in the first 20 min. However, proteases accumulated in the conditioned media up to 8 hr (Fig 1B). The release of all three proteases was dose-dependent and was detectable in supernatants of PMC stimulated with tc-LIG at concentrations 0.1 μM and higher (Fig 1C). PMC activation with 48/80 (0.5 mg/ml) induced the release of all three proteases in similar levels to 0.5 μM of tc-LIG (Fig. 1C).

*Nippostrongylus brasiliensis *antigen (*Nippo *Ag) (10 We/ml) induced no detectable release of any of the three proteases studied (Fig 1A). *Nippo *Ag-activated cells under these conditions released 10 ± 2% of β-hex; level similar with that released by 0.1 μM of tc-LIG (12 ± 2%), which however, was associated with protease release (Fig 1C). This amount of β-hex release was the highest we could obtain following PMC activation with *Nippo *Ag, under the conditions used in our experiments.

### PAR-2ap-induced proteolytic activity released from mast cells degrades TNF

**Figure 2:**
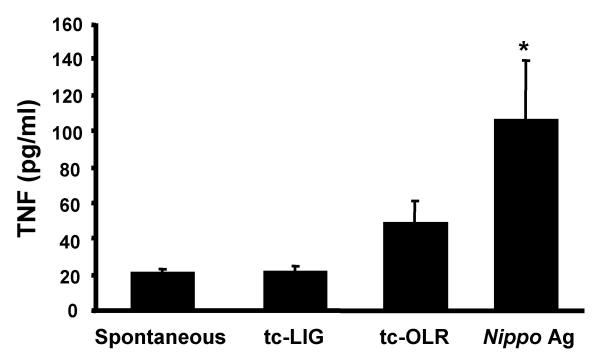
TNF release from PMC (1 × 10^6 ^cells) after 8 hr incubation with PAR-2ap (tc-LIG, 10 μM), PAR-2cp (tc-OLR, 10 μM) and Ag (10 We/ml). (Mean ± SEM, n = 4). Star indicates statistically significant difference from spontaneous (p < 0.05, n = 4).

On further comparing Fcε RI with tc-LIG-induced PMC activation we noted that Fcε RI-mediated activation induced TNF release while tc-LIG-mediated activation did not induce significant TNF release following PMC activation for up to 8 hr (Fig 2). One possible hypothesis to explain this effect was that TNF released by tc-LIG activated PMC was degraded by some of the proteases we showed to be released from mast cells at the same time.

**Figure 3:**
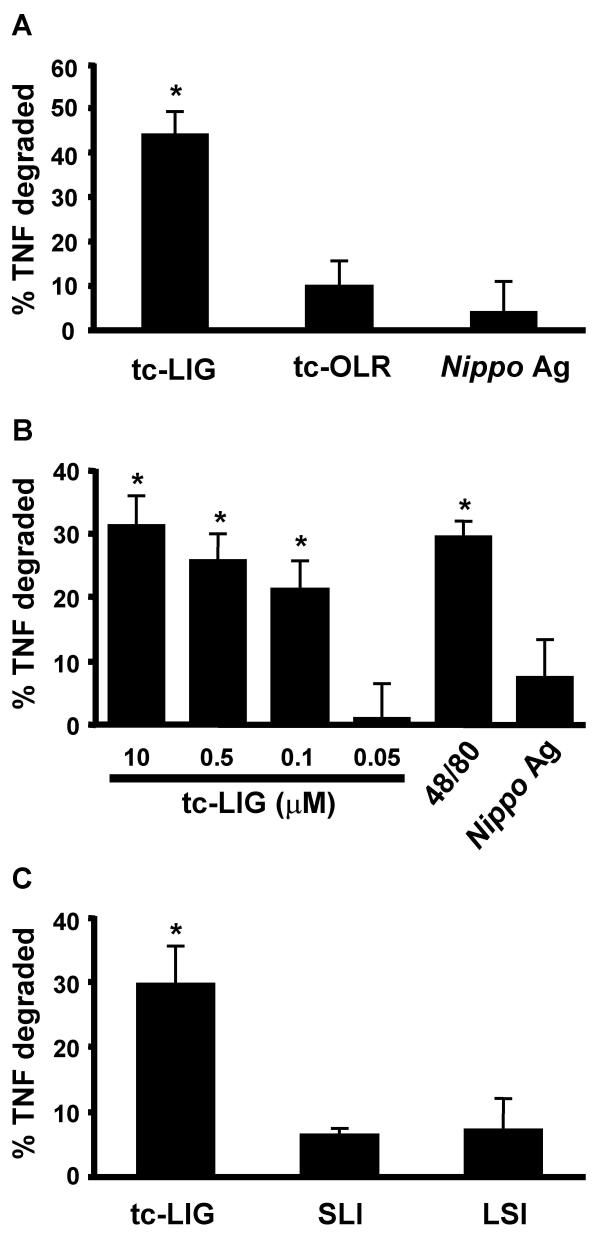
PAR-2ap, PAR-2cp compound 48/80 and Ag-mediated release of proteolytic activity from mast cells. (A) Supernatants from PMC treated with tc-LIG (10 μM), tc-OLR (10 μM), or Ag (10 We/mL) for 8 hr were incubated with 150 pg/mL of TNF. Proteolytic activity was calculated as % degraded TNF according to the formula given in Methods section. Values in the graph indicate proteolytic activity after the subtraction of activity released by sham-treated cells (17 ± 7 %). (B) TNF-degrading proteolytic activity released from PMC by various doses of tc-LIG (20 min). Values in the graph indicate proteolytic activity after the subtraction of activity released by sham-treated cells (22 ± 5 %). (C) TNF-degrading proteolytic activity released by SLI (PAR_2_-ap, 40 μM), LSI (PAR_2_-cp, 40 μM) and tc-LIG (10 μM) treated PMC (20 min). Values in the graph indicate proteolytic activity after the subtraction of activity released by sham-treated cells (23 ± 7 %). Values are shown as "mean ± SEM" (n = 3–5). Star indicates statistically significant difference from spontaneous (p < 0.05).

Therefore, we examined the ability of supernatants of PAR-2ap-activated PMC to degrade extracellular proteins. We used a bioassay for released protease activity, employing TNF as the cytokine to be degraded. PMC were incubated with no activators (sham treatment), tc-LIG (10 μM), tc-OLR (10 μM) or compound 48/80 (0.5 μg/ml) for 20 min or 8 hr at 37°C and the supernatants were collected. These supernatants or media were then seeded with 150 pg/ml of rat recombinant TNF and incubated for an additional 8 hr. At the end of the incubation TNF was measured by ELISA and the proteolytic activity was calculated as % degraded TNF (as discussed in the methods section). Proteolytic activity in the supernatants of sham-treated cells was subtracted from that in the supernatants of activated PMC. Supernatants from sham-treated PMC showed significant loss of seeded TNF (17 ± 7 % at 20 min and 22 ± 5 % at 8 hr) as compared to media. At both 20 min and 8 hr of treatment supernatants from tc-LIG-treated MC (10-0.1 μM) showed a greater loss of seeded TNF compared to supernatants of sham-treated MC (p < 0.05), suggesting tc-LIG-mediated activation induced the release of proteolytic activity. Proteolytic activity, following subtraction of spontaneous proteolytic activity released, was 44 ± 5 % at 8 hr and 30 ± 4 % at 20 min following PMC activation with 10 μM of tc-LIG (Fig. 3A and 3B respectively). Supernatants from tc-OLR- or *Nippo *Ag-treated cells showed no significant loss of TNF over that which occurred in sham-treated cells (Fig. 3A and 3B).

We also examined the ability of another PAR-2ap, SLI and its PAR-2cp, LSI, to release proteolytic activity from PMC (Fig. 3C). A small but significant increase in proteolytic activity over spontaneous release was induced by SLI (40 μM, 7 ± 1 %, p < 0.05). However, net SLI-mediated proteolytic activity released was not significantly different than that released by the inactive control peptide, LSI (40 μM, 7 ± 5 %).

**Figure 4:**
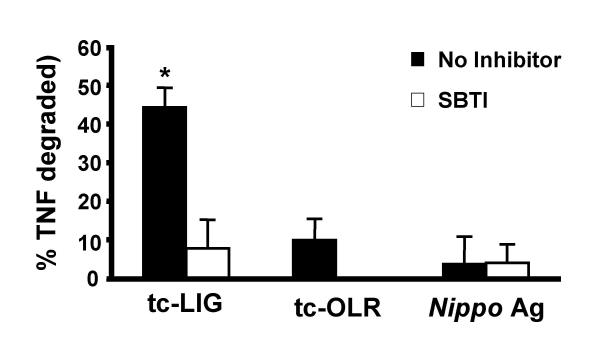
Effect of SBTI on the proteolytic activity in supernatants of tc-LIG-stimulated mast cells. Supernatants from PMC stimulated by tc-LIG, tc-OLR, and Ag for 8 hr were incubated with or without SBTI (1 mg/mL) before 150 pg/mL of TNF was added and % degradation calculated. Values indicate proteolytic activity after the subtraction of spontaneous release (17 % ± 7). Star indicates statistically significant difference from spontaneous (p < 0.05, n = 4–5). (Mean ± SEM).

To study whether tc-LIG-mediated TNF proteolytic activity was a result of serine protease activity, the supernatants were mixed with the broad spectrum serine protease inhibitor, SBTI (1 mg/ml) before seeding with TNF. SBTI inhibited TNF loss from the supernatants of tc-LIG (10 μM) stimulated PMC by 82% (Fig. 4), confirming that tc-LIG-induced loss of TNF was by the proteolytic activity of serine proteases.

## Discussion

We have previously shown that PMC express PAR-2 mRNA, that can be regulated by cytokines and PAR-2ap [13]. We have also shown that RMCP-1, RMCP-5 and CPA are stored in PMC and are the most prominent proteases produced by PMC [16]. In the present study we demonstrated that tc-LIG, a PAR-2ap, induces the release of RMCP-1, RMCP-5 and CPA from mast cells. Compound 48/80 induced comparable release of proteases form PMC, but FcεRI-mediated activation did not. We also showed that these, and possibly other proteases released at the same time from PMC, are capable of degrading TNF.

In this study, we provided the first direct evidence for serine protease release from PMC measured by Western blot analysis of the supernatants, in addition to proteolytic activity assays. The sizes of released RMCP-1 (~30 kDa), RMCP-5 (2 close bands, ~34 kDa) and CPA (3 close bands, ~41 kDa) are similar to the sizes of the stored forms of these proteases that we published previously [16]. The different bands for RMCP-5 and CPA are likely due to differential glycosylation, as has been shown before [16]. PAR-2 ap have been shown to release proteases from gastric pepsinogen secreting cells [17] and from epithelial cells [10]. A recent report showed release of tryptase from human colon mast cells following PAR-2ap-mediated activation [18]. In that case the concentration of PAR-2ap needed was higher than in our experiments and the effect was similar with FcεRI-mediated activation, while in our experiments proteases were released by PAR-2ap but not with *Nippo *Ag.

Previous studies have shown that activation of MC to release protease activity may be induced by a variety of agents both *in vivo *and *in vitro*. The release of RMCP-2 by rat mucosal mast cells has been reported to be induced by antigen challenge in parasitic infections, and during anaphylaxis [19-21]. The release of RMCP-2 mouse counterpart, MMCP-1, can be increased during parasitic infections [22]. Furthermore, rat CPA can be released by 48/80, Ca^2+ ^ionophore and antigen activation of PMC [23].

In our experiments FcεRI-mediated PMC stimulation did not release detectable levels of RMCP-1, RMCP-5 or CPA, or any proteolytic activity with the ability to degrade TNF. It is possible that FcεRI-mediated activation induces low levels of protease release which is undetectable by Western blotting. Furthermore, the lack of demonstrable proteolytic activity in the supernatants does not necessarily indicate that proteases are not released since it may be due to an FcεRI-mediated simultaneous release of protease inhibitors stored in the MC. Indeed, MC produce and release secretory leukocyte protease inhibitor (a chymase inhibitor) and latexin (a CPA inhibitor) [23,24]. Finally, FcεRI-mediated activation may selectively release proteases different from the ones released by 48/80 or PAR-2ap.

Given that RMCP-1 and RMCP-5 are present in the supernatants of tc-LIG-stimulated MC, it is likely that these proteases are involved in the TNF degradation. However, antibodies to RMCP-1 inhibited TNF degradation by supernatants of sham treated cells but did not affect the additional degradation of supernatants from tc-LIG activated MC (unpublished observation). We cannot rule out that PAR-2ap activated PMC release other proteases, including the tryptases RMCP-6 and RMCP-7 [25], which may contribute to TNF-degradation. It is also interesting that chymases and CPA, which are released in parallel, have synergistic effects [26]. Other proteases from leukocytes are known to be able to degrade TNF. These include cathepsin G [27], neutrophil elastase [28,29] and by proteases released from bacteria [39]. The same proteases can also degrade other cytokines, such as endothelin [31,32], lymphotoxin [27] and IFNγ [30].

Our data further suggest that MC may regulate TNF function by releasing proteases that can directly degrade this cytokine. Given that both TNF and serine proteases are stored and released from MC, our present findings suggest an important mechanism by which MC may regulate TNF function *in vivo*. It may be that such proteolytic activity directed against TNF, and possibly other cytokines, is an important anti-inflammatory function for mast cell serine proteases.

The expression of PAR-2 by mast cells and the involvement of MC in PAR-2-mediated inflammation has been controversial. *In vitro*, MC tryptase can stimulate histamine release by human tonsillar [33] and guinea pig [34] MC, but not from foreskin mast cells. The tryptase inhibitor APC366 inhibits IgE-dependent MC activation, and also inhibits calcium ionophore-induced histamine release [33]. Tryptase-mediated bronchoconstriction in sheep is histamine mediated [35], indicating that tryptase induces lung MC activation. PAR-2 has been identified on human [12] and rat [13] mast cells. MC have been implicated in rat paw oedema caused by PAR-2ap or trypsin administration [14], but other reports failed to confirm this observation [15]. Taken together these reports strongly suggest a role for tryptase and possibly PAR-2 in MC activation.

In a previous study we have shown that only one of two PAR-2ap (tc-LIG) activates β-hex release from PMC [13]. The other peptide, SLI, although it is a potent and selective PAR-2 agonist, was unable to induce release of β-hex as had also been shown before [36], although others showed that higher concentrations of SLI can induce the release of β-hex and to a greater extend histamine from rat PMC [37]. However, our study was the only one to use tc-LIG. In this study again only tc-LIG induced the release of proteases from PMC. SLI induced slightly increased release of proteolytic activity compared to sham treated cells but this release was not significantly different than the release induced by the control peptide LSI. We have previously shown that SLI is sensitive to proteases and its effects on MC increases in the presence of amastatin, an aminopeptidase inhibitor [13]. In contrast tc-LIG possesses a *trans-cinnamoyl *group, which acts to stabilize the peptide and prevent its degradation by aminopeptidases. It is unlikely that the different sensitivity to proteases can explain fully the difference between the effects of the two PAR-2ap peptides. It is also unlikely that the *trans-cinnamoyl *modification on tc-LIG is solely responsible for tc-LIG-mediated activation of MC, because it is also present on the reverse sequence peptide tc-OLR, but does not have the same effects with tc-LIG on protease release, as shown in this study, or in the release of β-hex, as we showed before.

Compound 48/80 along with other cationic compounds can activate MC by directly interacting with a pertussis toxin sensitive component [38]. Our previous work suggested that tc-LIG may activate MC through a 48/80-like mechanism, but appears to also possess a second mechanism of signalling that is distinct from that of 48/80 [13]. Thus, we cannot rule out the possibility that tc-LIG-mediated release of proteolytic activity may be mediated in part through a 48/80-like mechanism. Indeed, 48/80 induced similar levels of proteolytic activity and protease release to tc-LIG.

Recently, a new receptor activated by the PAR-2 activating peptide tc-LIGRLO-NH_2 _has been identified pharmacologically in murine vascular smooth muscle [39]. In that case, tc-LIG induced vasoconstriction, while the other PAR-2 activating peptide, SLI, did not have similar effects. The structure or the exact function of this receptor is not known. In our case also tc-LIG had a significant effect on protease release from mast cells while SLI had a very small effect. These data suggest that mast cells may express the same receptor as the one identified pharmacologically in smooth muscle cells.

## Conclusions

Our study provides evidence that a PAR-2ap, tc-LIG, activates MC to release proteases and proteolytic activity that could potentially have both pro- and anti-inflammatory functions. We further showed that these proteases may degrade extracellular proteins and affect the inflammatory environment in areas of mast cell activation. Although the presence and function of PAR-2 on MC is still controversial, our findings indicate that PAR-2 may be part of an autocrine loop. PAR-2 activation leads to the release of serine proteases which in turn may further activate more PAR-2 receptors on mast cells and also on other cells.

## Methods

### Reagents

Compound 48/80, 4-methylumbelliferyl-N-acetyl-β-D-glucosaminide (β-hexosaminidase (β-hex) substrate) and soybean trypsin inhibitor (SBTI) were purchased from Sigma Chemical Co. (St. Louis, MO). PAR-2ap and PAR-2 control peptides (PAR-2cp) were synthesized by the Peptide Synthesis Facility, Faculty of Medicine, University of Calgary. These peptides were determined to be ≥ 95 % pure by mass spectrometry and HPLC. Polyclonal RMCP-5 and CPA antibodies were produced and characterized as described previously [16]. Briefly, RMCP-5 (15 amino acids) and CPA (12 amino acids) NH_2_-terminal sequences were synthesized at Zymogenetics Inc, Seattle, WA, and used to immunize rabbits to develop specific polyclonal anti-protease antibodies. Professor H. Miller, Edinburgh, Scotland, kindly provided rabbit antibody to RMCP-1.

### Animal sensitization

Outbred male Sprague-Dawley rats (weight 250–500 g) were purchased from Charles River Canada Inc., (St. Constant, Quebec). Rats were maintained in an isolation room with filter-topped cages to minimize unwanted infections. For the experiments where MC were activated through their IgE receptor, rats were sensitized to *Nippostrongylus brasiliensis*, by a single subcutaneous injection of 3000 third-stage larvae in 0.5 mL of saline as described previously [40]. The experimental protocol was approved by the University of Alberta Animal Care Committee in accordance with the guidelines of the Canadian Council on Animal Care.

### Harvesting and enrichment of peritoneal mast cells

Fifteen mL of ice-cold Hepes-buffered (10 mM, pH 7.3) Tyrodes buffer supplemented with 0.1% BSA was injected into the peritoneal cavity of each rat for the isolation of PMC. MC in peritoneal lavage fluids were enriched by centrifugation through a discontinuous density gradient of Percoll, as described previously [41]. Recovered MC were >95 % pure. Cell viability was >97 %.

### Mast cell activation

After isolation and enrichment, PMC were rested in RPMI (Invitrogen, Burlington, Ontario) supplemented with 5% FBS for 2 hr at 37°C. After incubation, the cells were washed twice by centrifugation (150 g) and resuspended in RPMI at 1 × 10^6 ^cells/mL. Cells were placed in 1.5 mL Eppendorf tubes or in 48 well plates, incubated at 37°C for 10 min, and then the same volume of pre-warmed (37°C) PAR-2ap or controls in complete RPMI were added, to give a final cell concentration of 0.5 × 10^6 ^cells/mL. The cells were incubated for different times (20 min to 8 hr) depending on the experiment.

To measure spontaneous release of mediators by PMC, cells were mixed with media alone. As positive controls, either compound 48/80 (0.5 μg/mL) or *Nippostrongylus brasiliensis *Antigen (1–100 worm equivalents (WE)/mL [40]) were mixed with cells under the same conditions. After incubation, tubes were placed on ice for 10 min and then centrifuged (150 g) to separate supernatant from cells. The supernatants were collected in tubes and the same volume of fresh media was added to the pellets, which were then resuspended. Cell viability was assessed at different times. Cell pellets and supernatants were stored at -70°C until assayed for their content of cytokines or proteolytic activity.

### SDS-PAGE and western blot analysis

Supernatants were concentrated (10×) using Centricon (YM-10) centrifugal filter devices (Millipore, Bedford, MA). For Western blot analysis, proteins were transferred electrophoretically (25 V, 35 min) to a polyvinylidene difluoride (PVDF) membrane (Bio-Rad Laboratories, Mississauga, ON) using the Semi-Dry Trans Blot System. The membranes were blocked in Tris-buffered saline containing 0.02% Tween, 5% w/vmilk (Bio-Rad Laboratories) and 5 % v/vgoat serum (Invitrogen) for 1 hr. The membranes were probed with 1/1000 dilution of anti-RMCP-1, 1/600 anti-CPA, 1/5000 anti-RMCP-5 and then incubated with donkey anti-rabbit IgG HRP-conjugated antibody (1:5000). Protein bands were detected by enhanced chemiluminescence using ECL Western blotting detection system (Amersham Pharmacia Biotech, Quebec, Canada).

### β-hexosaminidase (β-hex) assay

β-hex was measured in the supernatants and cell pellets, as described [42]. Results are expressed as β-hex released as a percent of total β-hex (pellet + supernatant). Values shown have been corrected for the spontaneous β-hex release.

### Proteolytic activity assay and protease inhibition assay

To measure release of proteolytic activity, supernatants from stimulated PMC (containing secreted TNF) were transferred to a 96-well plate. After 2 min incubation, exogenous TNF or medium was added to the supernatants and mixed to give a final concentration of 150 pg/ml. Plates were incubated at 37°C for 8 hr and then TNF content was measured by ELISA. Percent TNF proteolysis was calculated by the following formula:

% TNF degraded = 1 - (TNF recovered / (rat recombinant TNF seeded + measured TNF release) × 100)

For protease inhibition experiments SBTI (1 mg/ml) was added to the supernatants before seeding with TNF. The supernatants were then processed as above and used to measure TNF degradation.

### TNF measurements

Supernatants from activated PMC were analysed for TNF using a rat TNF ELISA kit (Endogen, Woburn, MA), according to manufacturer's instruction. The sensitivity of the TNF assay was < 10 pg/ml. To exclude the possibility that proteases contained in PMC supernatants interfere with ELISA determination of TNF we incubated the TNF antibody coated wells with PMC supernatants washed them and then added specified amounts of TNF for determination. Pre-incubation with PMC supernatants did not affect the ability to measure TNF, indicating that the proteases in PMC supernatants do not degrade the antibodies of the assay.

### Statistics

All values are given as mean ± standard error of mean (SEM) for the numbers of experiments noted and statistical analyses were performed using the Student's *t*-test and ANOVA.

## Abbreviations

β-hex β-hexosaminidase

CPA carboxypeptidase-A

LSI LSIGRL-NH_2 _(PAR2-cp)

MC mast cell

PAR protease-activated receptor

PAR-ap protease-activated receptor-agonist peptide

PAR-cp protease-activated receptor-control peptide

PMC peritoneal mast cells

RMCP-1,5 rat mast cell protease-1, 5

SBTI soybean trypsin inhibitor

SLI SLIGRL-NH_2 _(PAR2-ap)

tc-LIG *trans-cinnamoyl*-LIGRLO-NH_2_

tc-OLR *trans-cinnamoyl*-OLRGIL-NH_2_

*Nippo *Ag *Nippostrongylus brasiliensis *Antigen

WE worm equivalent**.**

## Authors' contributions

HNA carried out the majority of the experiments presented and drafted the manuscript. This work was part of his MSc thesis. GRS assisted with mast cell isolation and some of the activation experiments and participated in the experimental design. JLW participated in the design of the study. MDH participated in the design of the study. ADB participated in the design of the study, supervised the work shown and made substantial contributions in manuscript preparation. HV participated in the design of the study and contributed in the preparation of the final manuscript. All authors read and approved the final manuscript.
